# Growth Performance and Hematological Changes in Growing Sika Deers Fed with Spent Mushroom Substrate of *Pleurotus ostreatus*

**DOI:** 10.3390/ani12060765

**Published:** 2022-03-18

**Authors:** Chongshan Yuan, Min Wu, Xinyuan Chen, Changze Li, Aiwu Zhang, Wenfa Lu

**Affiliations:** College of Animal Science and Technology, Jilin Agricultural University, Changchun 130118, China; 18844146800@163.com (C.Y.); koo331500@163.com (M.W.); chenxy9797@163.com (X.C.); mbconfuse@163.com (C.L.)

**Keywords:** average daily weight gain, blood index, digestibility, growing sika deer, spent mushroom substrate

## Abstract

**Simple Summary:**

With the rapid development of the mushroom industry, a large number of spent mushroom substrate (SMS) has also been produced. SMS can be easily digested by ruminants and is suitable for feeding animals, such as cows, sheep, as well as deer. The results of this study show that the dietary spent mushroom substrate of *Pleurotus ostreatus* (SMS-MP) has no obvious effect on the physiological condition of growing sika deer, at the same time it can reduce the cost of feeding and avoid environmental pollution caused by improper disposal of SMS-MP.

**Abstract:**

The purpose of this experiment is to expand the feed of growing sika deer and to explore the effects on growing sika deer of the spent mushroom substrate of *Pleurotus ostreatus* (SMS-MP). Twelve immature female growing sika deer were randomly assigned to four groups. The ratios of SMS-MP to replace concentration supplements were 0%, 10%, 20%, and 30%, respectively, and the growth performance, feed intake and apparent digestibility, serum biochemical indexes, blood physiological indexes, serum immune globulin and plasma amino acid of growing sika deer were measured. The results of the current study confirmed the applicability of SMS-MP as a feed ingredient in growing sika deer diets. There was no significant change in growth performance and hematology of growing sika deer when the concentrate supplement was replaced with 10–20% SMS-MP. However, replacing 30% of concentrate supplements with SMS-MP in the growing sika deer diet resulted in significantly decreased Hb and HCT levels. It can be concluded that, as a waste resource, adding a small amount of SMS-MP has no significant effect on the growth of sika deer, and at the same time can reduce the consumption of concentrate supplements, thereby improving the economic benefits of sika deer breeding.

## 1. Introduction

With the increase in global mushroom production [1], the production of waste spent mushroom substrate (SMS) as a by-product of mushroom cultivation has been growing rapidly [2]. In Asia, one of the major problems in mushroom production is the treatment and disposal of the spent mushroom substrate (SMS) which is a co-product of the mushroom industry [3,4,5]. Recently, there has been increasing public concern on the effects of SMS disposal on the environment. It would be more economical and beneficial if SMS could be recycled and reused. SMS could potentially be a source of planting industry, such as accelerating the maturation of compost [6], enhancing soil organic matter and nutrient contents [7], and increasing the morphological growth of tomato and pepper seedlings [8]. SMS is an organic by-product rich in protein, polysaccharides, which also contains several bioactive compounds resulting from fungal growth, such as vitamins and some trace elements such as Fe, Ca, Zn and Mg, and therefore can potentially be a value-added product. One of the recycling methods is to use it as animal feed since the ingredients used as SMS are used as animal feeds as well. Many studies have already been performed in regards to the advantages and processing methods of feed based on SMS [9,10]. SMS has been widely reported to be used in feed processing [11] and animal feed presently [12,13,14]. SMS can be easily digested by ruminants and are suitable for feeding animals, such as cows [15] and sheep [16]. Furthermore, SMS is considered to be an animal feed with nutritional value [17]. One of the more popularly cultivated mushroom species worldwide belongs to the genus *Pleurotus* *ostreatus,* it can be cultivated on a variety of substrates such as rice straw, wheat straw, corncob, cottonseed hull as well as other substrates [18]. *Pleurotus* *ostreatus* increase the nutritional content of SMS during growth, at the same time, different components of SMS also affect the content of mushroom nutrients [19]. Most sika deer in China are fed in captivity. According to ancient Chinese medicine monographs, sika deer antler has long been used as a traditional tonic or alternative medicine [20]. At the same time, raising female sika deer is very important to expand the group size and increase the scale of antler production. To our knowledge, there are no published reports on using SMS as a diet for sika deer. This study is designed to evaluate the effectiveness of dietary spent mushroom substrate of *Pleurotus ostreatus* (SMS-MP) on the average daily weight gain, blood index, digestibility of female growing sika deer in order to reduce breeding costs while avoiding environmental pollution caused by improper disposal. 

## 2. Materials and Methods

### 2.1. Animals, Diets, and Experimental Designs

Twelve healthy immature female growing sika deer with the similar initial body weight (*P* > 0.05) were randomly assigned to four groups (three deer / group), living in the artificial feedlot with no grass, tree canopy cover, etc. The four groups were named I, II, III and IV, respectively, in which a different experimental diet was given to each group. Three levels of corncob SMS-MP (dried in the open air) were adopted in the four groups as follows: group 1 (I): basal diet, without substitute of SMS-MP; group 2 (II): replace 10% of concentrate with SMS-MP; group 3 (III): replace 20% of concentrate with SMS-MP; group 4 (IV): replace 30% of concentrate with SMS-MP. Each group was fed concentrate supplement with the same weight and offered three times per day at 3:30, 10:30, 16:30. Silage and water was *ad libiturn*. The growth trial lasted for 31 days according to a randomized design, during which feed intake was recorded daily, and the feed efficiency and the average daily gain (ADG) were calculated. The composition and nutrition of concentrate supplement are presented in Table 1, and the chemical composition of feeds are presented in Table 2. The main component of SMS-MP was corncob. The chemical composition of dried SMS-MP is presented in Table 3. 

### 2.2. Sample Collection and Treatment

#### 2.2.1. Samples of Silage, Concentrate Supplement and Feces

Silage, concentrate supplement and feces were weighed and recorded daily. Samples of silage, concentrate supplement and feces were dried in a forced-air oven at 65 °C for at least 48 h until sub-samples reached constant weight, and then ground in a Wiley mill to pass a 2 mm screen, prepare for preservation until laboratory analysis.

#### 2.2.2. Blood Sampling

On the morning of the end of the trial, all of the growing sika deer were anesthetized with xylazine hydrochloride, which was administered using a blow gun-dart syringe at a dosage of 1 mL per deer. Then, 5 mL of blood was collected with vacuum tubes (Vacutainer; Becton Dickinson and Co. Rutherford, NJ, USA) containing sodium heparin, 2 mL of sample was taken for the detection of blood physiological indexes, and the other 3 mL samples were centrifuged at 3000 rpm to take the plasma to test the amino acids. Another 5 mL of whole blood was put into a vacuum tube, and the serum obtained from whole blood was used to detect serum biochemical indexes and immunoglobulins. Samples were stored at −20 °C for later analysis. After sample collection, the sika deer were injected with 1 mL Nikethamide immediately to wake up the growing sika deer.

### 2.3. Determination 

#### 2.3.1. Body Weight Gain Determination

Body weight gain was obtained by the difference in weight between the first day and the last day of the experiment.

#### 2.3.2. Feed Intake and Nutrient Digestibility

Feed intake was recorded daily by weighing the silage and concentrate complement offered, and the refusals of the previous day. In the last 8 days of the experiment, samples of silage, concentrate supplement and feces were collected per day.

Dry matter (DM) content of concentrate supplement, silage and feces samples was determined in a forced-air oven at 105 °C to constant weight. N (CP = N × 6.25) determinations were carried out using the LECO FP-528N/Protein tester (LECO, Corporation). Ether extract (EE) and crude fiber (CF) were analyzed in duplicate as extraction disappearance in anhydrous diethyl ether using Official Analytical Chemists (AOAC, 1998) [21]. Gross energy concentration in feces and feedstuffs was determined using an adiabatic bomb calorimeter (Changji, Shanghai, China). Ash was determined after thermal decomposition in a muffle furnace at 525 °C for 12 h, and organic matter (OM) was determined by difference.

A nutrient digestibility trial was carried out in which acid insoluble ash was used as an internal indigestibility marker, and digestibility coefficients of digestion were calculated according to the following equation:

(1) $$N=100-100\times \frac{D}{F}\times \frac{D_{1}}{F_{1}}$$

where *N* represents apparent digestibility of nutrients, *D* is the content of indicator in the diet, *F* is the content of indicator in the feces, *F*_1_ is the content of nutrition in the feces; *D*_1_ is the content of nutrition in the diet.

#### 2.3.3. Analysis of Blood

Plasma-free amino acid was determined using high-performance liquid chromatography on an L3000 AA Analyzer (Shimadzu Ltd., Kyoto, Japan). Additional procedural details are described elsewhere [22].

The concentration of albumin (ALB), total protein (TP), globulin (GLO), calcium (Ca), glucose (GLU), urea nitrogen (BUN), amylase (AMY), cholesterol (CHOL), alanine aminotransferase (ALT), total bilirubin (TBIL), alkaline phosphatase (ALP), inosine (CRE), urea nitrogen (BUN), creatine kinase (CK) in serum were analyzed by BC-5300vet automatic blood cell analyzer. 

The values of white blood cells (WBC), red blood cells (RBC), hemoglobin (Hb), hematocrit (HCT), MCV (Mean erythrocyte volume), mean corpuscular hemoglobin (MCH), mean corpuscular-hemoglobin concentration (MCHC), variation of RBC distribution width (RDW-CV) were determined using a hematological analyzer (Sysmex K-1000, TAO Medical Electronics Co., Kobe, Japan).

IgA, IgG and IgM of serum were determined using an enzyme-linked immunoassay kit (Shanghai Enzyme Biotechnology Co., Ltd., Shanghai, China), each index corresponding to a specific kit, according to the manufacturer’s instructions. 

### 2.4. Statistical Analysis

Statistical analysis was via least-squares analysis of variance (ANOVA), following the general linear models procedure of SPSS (SPSS 19.0 for Windows; SPSS Inc., Chicago, IL, United States). Each experiment was repeated at least three times. Data are presented as the mean ± SD. The variances were first analyzed using a homogeneity test. If the data met the assumption of homoscedasticity, the significance of differences in the means was determined by one-way ANOVA and subsequent LSD test. *P* < 0.01 was considered as greatly significant; *P* < 0.05 was considered as significant.

## 3. Results

### 3.1. Effects on Growth

From Table 4, we can see that there is no difference in IW, FW and ADG (*P* > 0.05). It can also be seen that there are no differences in F/W and E/W among the four groups (*P* > 0.05).

### 3.2. Effects on Feed Intake and Apparent Digestibility 

There were no differences in nutrient intake (*P* > 0.05) during the whole experiment. It can also be noted that there were no differences in apparent digestibility of EE, CP and DM among the four groups. Furthermore, the digestibility of OM in group I was greater than that in group III (*P* < 0.05) as indicated in Table 5. 

### 3.3. Effects on Serum Biochemical Indices

Based on the serum biochemical indexes of the tested deer (Table 6), SMS-MP silage had no significant effects on the serum concentrations of AST, BUN, CRN, ALP, ALB, TP or ALT (*P* > 0.05). Serum concentrations of γ-GT in group I was greater than that in other groups (*P* < 0.05). GLUC in group III was significantly higher than group II and I (*P* < 0.05), and extremely significantly higher than that of group IV (*P* < 0.01).

### 3.4. Effects on Blood Physiological Indexes 

The parameters of serum physiological indexes are shown in Table 7. RBC and Hb in IV were significantly lower than in other groups (*P* < 0.05). 

### 3.5. Effects on Serum Immune Globulin

There were no differences in IgA and IgG during the whole experiment. Higher content of IgM was observed with the substitution of SMS for 30% of the concentrate supplement (*P* < 0.01) as indicated in Table 8.

### 3.6. Effects on Plasma Blood Amino Acid

Effects of SMS-MP on Blood amino acids are shown in Table 9. Parameters of blood amino acid were not affected by dietary SMS-MP level (*P* > 0.05). 

## 4. Discussion

The present experiments were designed to explore the effect of adding SMS-MP to concentrate supplements on the growth performance of growing sika deer. Moreover, adding a small amount of SMS-MP to concentrate supplements had no effect on the health of growing sika deer.

Above all, there was no significant difference in the ADG of growing sika deer in each group, meanwhile, the ratio of F/W in the control group was higher than other groups, but was not significantly different. Thus, it can be concluded that SMS-MP is conducive to the growth of sika deer, improving the utilization rate of feed and reducing the cost of breading. The feed intake of sika deer can be affected by both physiological factors and external factors, including dietary habits, lifestyle behaviors, physiological factors, environmental factors and animal learning abilities [23]. The biodegradation of straw with *Pleurotus ostreatus* increased its nutritional value and digestibility in ruminant diets [24]. According to this study, compared with the control group, SMS-MP had no effect on feed intake of dry matter and organic matter, the apparent digestibility of EE, meanwhile, CP and DM had no effect on growing sika deer. The findings of the current study support that of previous research which determined that SMS-MP could be used as ruminant feed without any harmful effects on eating behavior in cattle [14]. In this study, we found that the CP in concentrate supplements would decrease with the addition of SMS-MP, and the addition of a small amount of SMS-MP did not affect growing sika deer, indicating that the protein consumption of growing sika deer can be reduced, thereby avoiding environmental pollution caused by excessive consumption of N elements. However, in this study, the SMS-MP replacement of 20% reduces the digestibility of OM, which may be caused by the low organic content of SMS-MP compared with the concentrate supplement and further research is needed. 

In ruminants, blood BUN can be influenced by the quantity of dietary protein, level of feed intake, and protein degradability in the rumen [25]. Blood glucose level is used as an indicator of ruminant energy status. In the current study, sika deer replacement of 30% with SMS-MP had lower glucose concentration, and this result may be attributed to lower energy consumption as compared to other groups. There was no significantly different level, however, higher BUN concentration was founded in group IV, it might be concluded that, higher nitrogen (N) due to replacement of 30% with SMS-MP. As an ectoenzyme, γ-GT plays a key role in GSH homeostasis by breaking down extracellular GSH and providing cysteine [26]. γ-GT from mammals have been found to play roles in cell defense against oxidative stress, Parkinson’s disease, other neurodegenerative diseases, and cardiovascular disease [27,28]. Compared with other groups, a remarkable increase in the concentration of γ-GT was observed for replacement of 10% with SMS-MP in concentrate supplement. It showed that a small amount of SMS-MP in diets can improve the antioxidant capacity of growing sika deer. Jae et al [29] identified that Hb and HCT levels in elk-fed diets with SMS were significantly increased. As the results showed that the contents of Hb and HCT increased with SMS feeding, but were not significantly different. Regardless of that, replacing 30% of concentrate supplements with SMS-MP in the growing sika deer diet may result in decreased Hb and HCT levels significantly. It means that the addition of high content of SMS-MP may have an adverse effect on the growth of sika deer.

IgM is the first antibody isotype to appear during evolution, ontogeny and immune responses. IgM not only serves as the first line of host defense against infections but also plays an important role in immune regulation and immunological tolerance [30]. Natural IgM autoantibodies (IgM–NAA) are rapidly produced to inhibit pathogens and abrogate inflammation mediated by invading microorganisms and host neoantigens [31]. As reported by Rahman et al. [32], oxidative stress could be inhibited by IgM anti-MDA. Moreover, it is reported that supplementation of cordyceps militaris spent mushroom substrate at 2 g/kg of diet increases immunoglobulin secretion of growing pigs [33]. The findings of our current study imply that SMS-MP inclusion in the diet may have modulating effects on the immune system by activating monocytes, although no significant differences were seen between the SMS-MP groups and control.

Blood or plasma-free amino acid pools represent only a very small proportion of tissue-free amino pools. Plasma-free amino acid profiles have been used as a tool to study nutritional and metabolic aspects of protein status. Static measurements of plasma amino acid levels have limited value as sensitive indicators of protein status [34]. Determination of metabolite or nutrient levels in readily available body fluids (blood and urine) is a long-established practice in studying metabolic changes in intact animals [35]. Plasma alpha amino nitrogen and free amino acid (AA) levels have been determined in an effort to assess protein status more clearly. The sum total of all factors influencing total body AA flux, that is protein synthesis, protein degradation, tissue uptake and efflux (including the role of hormones or dietary factors on these processes) and influx from the small intestine and AA catabolism are reflected by the concentration of AA in the plasma [36]. In this study, there was no significant difference in plasma amino acid content between the groups (*P* > 0.05). It can be concluded that SMS-MP has no significant effect on the plasma amino acids in the sika deer.

Our previous unpublished study found that replacement of SMS-MP in adult male sika deer and sika doe concentrate supplements for 4 and 11 months, respectively, had no effect on their health. It can be indicated that SMS-MP can be safely replaced with the concentrate supplements of growing sika deer for a long time. The breeding scale of sika deer in China is very small. Due to the actual operation restrictions of the experiment, we can only select a small number of samples (three deer/group) for preliminary experiments. Our results preliminarily indicated that a small amount of SMS-MP can be replaced with the concentrate supplements of growing sika deer. Later, it needs to be extended to larger groups to observe the effect of SMS-MP on growing sika deer.

## 5. Conclusions

The results of the current study confirmed the applicability of SMS-MP as a feed ingredient in growing sika deer diets. A quantity of 10% and 20% with SMS-MP can be safely replaced with the concentrate supplement of growing sika deer. The economic benefits of growing sika deer breeding can be improved by making full use of SMS-MP to reduce the consumption of concentrate supplements.

## Figures and Tables

**Table 1:** Ingredients of concentrate supplement (%).

Ingredients	Percentage
Corn	78.55
Soybean meal	20.00
NaCl	1.20
Stone powder	0.15
Ca(HCO_3_)_2_	0.10

**Table 2:** Chemical composition of dried concentrate supplements (%, DM).

Parameter	I	II	III	IV
Gross Energy (GE, MJ/kg)	15.87 ± 0.78	15.79 ± 0.83	15.39 ± 0.54	15.19 ± 0.35
Organic matter (OM)	96.04 ± 0.04	97.03 ± 0.12	95.75 ± 0.07	95.23 ± 0.16
Ether extract (EE)	16.57 ± 1.26	16.23 ± 0.60	15.93 ± 1.03	15.29 ± 0.47
Crude protein (CP)	9.16 ± 0.19	8.89 ± 0.08	7.70 ± 0.31	6.25 ± 0.12

**Table 3:** Chemical composition of dried SMS-MP (%, DM).

Parameter	SMS-MP
GE (MJ/kg)	14.79 ± 1.07
OM	93.23 ± 0.33
EE	9.13 ± 0.96
CP	3.01 ± 0.82
Crude fiber (CF)	31.70 ± 1.07
Phosphorus (P)	0.22 ± 0.01
Calcium (Ca)	1.46 ± 0.59

**Table 4:** Effects of dietary supplementation with different inclusion of SMS-MP on growth performance of growing sika deer.

Items	I	II	III	IV
initial weight (IW, kg)	53.27 ± 2.65	52.02 ± 3.73	51.80 ± 3.59	52.35 ± 2.63
final weight (FW, kg)	57.03 ± 1.81	56.03 ± 4.21	54.35 ± 3.71	56.23 ± 2.08
average daily weight gain (ADG, g)	125.56 ± 57.38	133.89 ± 17.82	119.17 ± 60.10	129.44 ± 30.97
weight gain to feed ratio (F/W, %)	10.17 ± 4.08	7.91 ± 1.13	9.51 ± 4.45	9.16 ± 1.94
net energy/weight gain (E/W, %)	139.63 ± 39.85	124.89 ± 17.99	123.67 ± 28.99	139.18 ± 29.54

**Table 5:** Effects of dietary supplementation with different inclusion of SMS-MP on feed intake and apparent digestibility of growing sika deer.

Items	I	II	III	IV
Dry matter intake (g·d^−1^)	1126.95 ± 176.51	1065.67 ± 216.43	1114.36 ± 131.39	1125.29 ± 164.35
Organic matter intake (g·d^−1^)	1071.88 ± 166.25	1019.73 ± 203.77	1058.39 ± 123.76	1065.76 ± 154.80
Digestibility of OM (%)	70.23 ± 6.94 ^a^	68.15 ± 9.10 ^ab^	63.01 ± 5.12 ^b^	66.60 ± 10.60 ^ab^
Digestibility of EE (%)	79.23 ± 4.52	80.41 ± 5.23	77.37 ± 3.40	77.00 ± 7.60
Digestibility of CP (%)	68.46 ± 8.57	64.92 ± 14.96	69.56 ± 9.85	72.90 ± 10.79
Digestibility of DM (%)	68.85 ± 6.82	65.48 ± 9.06	61.56 ± 4.99	65.74 ± 10.01

Note: ^a, b^ Values within a row with different superscripts differ significantly (*P* < 0.05).

**Table 6:** Effects of dietary supplementation with different inclusion of SMS-MP on serum biochemical indexes of growing sika deer.

Items	I	II	III	IV
AST (U/L)	51.67 ± 7.64	54.67 ± 4.62	54.67 ± 1.53	49.67 ± 2.08
BUN (mmol/L)	4.72 ± 0.59	5.29 ± 1.45	4.70 ± 1.00	6.45 ± 1.27
CRE (umol/L)	137.00 ± 4.36	137.33 ± 10.02	136.00 ± 15.72	141.00 ± 7.81
γ-GT (U/L)	19.33 ± 3.21 ^b^	25.67 ± 4.04 ^a^	19.33 ± 2.31 ^b^	21.67 ± 2.08 ^b^
GLUC (mmol/L)	6.99 ± 0.99 ^ABb^	7.28 ± 1.31 ^ABb^	9.99 ± 1.31 ^aA^	6.31 ± 0.74 ^cB^
ALP (U/L)	238.00 ± 44.19	354.67 ± 68.97	313.00 ± 149.92	228.33 ± 72.59
ALB (g/L)	24.33 ± 0.58	25.33 ± 0.58	25.00 ± 1.73	25.33 ± 0.58
TP (g/L)	54.60 ± 1.42	55.67 ± 0.55	54.53 ± 4.01	55.03 ± 1.07
ALT (U/L)	54.00 ± 22.72	60.00 ± 4.58	54.33 ± 6.66	57.33 ± 4.04

Note: ^a, b^ Values within a row with different superscripts differ significantly (*P* < 0.05). ^A, B^ Values within a row with different superscripts differ extremely significantly (*P* < 0.01). AST: aspartate aminotransferase; BUN: blood urea nitrogen; CRE: creatinine; γ-GT: gamma-glutamyl transpeptidase; GLUC: blood sugar; ALP: alkaline phosphatase; ALB: albumin; TP: total protein; ALT: alanine aminotransferase.

**Table 7:** Effects of dietary supplementation with different inclusion of SMS-MP on blood physiological indexes of growing sika deer.

Items	I	II	III	IV
WBC (10^9^/L)	2.97 ± 1.57	3.01 ± 1.01	3.28 ± 1.44	1.68 ± 0.84
RBC (10^12^/L)	6.79 ± 0.63 ^a^	6.71 ± 0.50 ^a^	7.92 ± 1.61 ^a^	5.79 ± 0.40 ^b^
Hb (g/L)	92.67 ± 4.73 ^a^	99.33 ± 4.51 ^a^	103.33 ± 15.53 ^a^	81.00 ± 8.72 ^b^
HCT (%)	0.51 ± 0.23	0.63 ± 0.07	0.43 ± 0.14	0.46 ± 0.22
MCV (fL)	74.93 ± 32.01	93.20 ± 11.79	58.73 ± 33.75	79.17 ± 36.83
MCH (pg)	13.70 ± 0.95	14.60 ± 0.44	13.13 ± 0.84	14.00 ± 1.15
MCHC (g/L)	215.00 ± 112.41	158.00 ± 16.52	263.33 ± 103.37	210.00 ± 108.86
RDW-CV (%)	0.21 ± 0.01	0.22 ± 0.01	0.22 ± 0.01	0.20 ± 0.01

Note: ^a, b^ Values within a row with different superscripts differ significantly (*P* < 0.05). WBC: white blood cell; RBC: red blood cell; Hb: hemoglobin; HCT: hematocrit; MCV: mean red blood cell volume; MCH: mean corpuscular hemoglobin; MCHC: mean red blood cell hemoglobin concentration; RDW-CV: coefficient of variation of red blood cell distribution width.

**Table 8:** Effects of dietary supplementation with different inclusion of SMS-MP on serum immune globulin of growing sika deer (mg/mL).

Items	I	II	III	IV
IgA	28.72 ± 10.53	41.59 ± 6.84	41.01 ± 6.38	28.75 ± 9.32
IgG	47.15 ± 16.84	20.68 ± 0.54	47.52 ± 20.88	44.50 ± 49.80
IgM	1.97 ± 0.10 ^B^	2.96 ± 0.16 ^B^	2.84 ± 0.02 ^B^	3.12 ± 0.49 ^A^

Note: ^A, B^ Values within a row with different superscripts differ extremely significantly (*P* < 0.01). IgA: Immunoglobulin A; IgG: Immunoglobulin G; IgM: Immunoglobulin M.

**Table 9:** Effects of dietary supplementation with different inclusion of SMS-MP on plasma amino acid of growing sika deer (µmol/mL).

Items	I	II	III	IV
Ala	28.32 ± 17.17	21.11 ± 0.54	20.41± 7.29	22.48 ± 2.52
Gly	690.29 ± 228.19	637.32 ± 71.88	678.78 ± 156.98	669.74 ± 58.88
Ile	162.04 ± 66.29	109.80 ± 31.29	99.86 ± 37.55	132.25 ± 11.00
Leu	101.60 ± 53.48	71.69 ± 9.02	73.04 ± 38.69	86.14 ± 5.24
Met	75.60 ± 16.53	82.07 ± 17.13	67.91 ± 42.62	106.60 ± 4.90
Phe	63.63 ± 13.03	70.78 ± 1.99	63.60 ± 9.25	72.60 ± 2.01
Pro	1.79 ± 0.50	1.67 ± 0.08	1.84 ± 0.77	1.55 ± 0.32
Ser	2.46 ± 0.44	3.06 ± 0.13	2.98 ± 0.94	3.11 ± 0.07
Thr	6.37 ± 1.34	7.82 ± 0.05	8.39 ± 5.47	7.91 ± 0.10
Tyr	0.62 ± 0.25	0.90 ± 0.00	1.42 ± 1.67	0.90 ± 0.00
Val	117.96 ± 1.69	121.11 ± 1.78	132.14 ± 16.00	118.05 ± 1.97

Note: Ala: alanine; Gly: Glycine; Ile: Isoleucine; Leu: Leucine; Met: methionine; Phe: Phenylalanine; Pro: Proline; Ser: serine; Thr: threonine; Tyr: tyrosine; Val: valine.

## Data Availability

The datasets generated and/or analysed during the current study are available from the corresponding author on reasonable request. And they will be provided during review.
